# L(3)mbt and the LINT complex safeguard cellular identity in the *Drosophila* ovary

**DOI:** 10.1242/dev.160721

**Published:** 2018-04-04

**Authors:** Rémi-Xavier Coux, Felipe Karam Teixeira, Ruth Lehmann

**Affiliations:** 1 Howard Hughes Medical Institute (HHMI) and Kimmel Center for Biology and Medicine of the Skirball Institute, Department of Cell Biology, New York University School of Medicine, New York, NY 10016, USA; 2 Department of Genetics, University of Cambridge, Downing Street, Cambridge CB2 3EH, UK

**Keywords:** L(3)mbt, Lint-1, Tissue identity, Nanos, Oogenesis, *Drosophila*

## Abstract

Maintenance of cellular identity is essential for tissue development and homeostasis. At the molecular level, cell identity is determined by the coordinated activation and repression of defined sets of genes. The tumor suppressor L(3)mbt has been shown to secure cellular identity in *Drosophila* larval brains by repressing germline-specific genes. Here, we interrogate the temporal and spatial requirements for L(3)mbt in the *Drosophila* ovary, and show that it safeguards the integrity of both somatic and germline tissues. *l(3)mbt* mutant ovaries exhibit multiple developmental defects, which we find to be largely caused by the inappropriate expression of a single gene, *nanos*, a key regulator of germline fate, in the somatic ovarian cells. In the female germline, we find that L(3)mbt represses testis-specific and neuronal genes. At the molecular level, we show that L(3)mbt function in the ovary is mediated through its co-factor Lint-1 but independently of the dREAM complex. Together, our work uncovers a more complex role for L(3)mbt than previously understood and demonstrates that L(3)mbt secures tissue identity by preventing the simultaneous expression of original identity markers and tissue-specific misexpression signatures.

## Abstract (precis)

**Highlighted Article:** Characterization of the developmental defects of *l(3)mbt* mutant ovaries shows that L(3)mbt regulates tissue-specific gene signatures to secure the identity of somatic ovarian and germline tissues.

## INTRODUCTION

Development requires tight control of gene expression as differentiating cells must express lineage-specific genes while repressing genes that promote other fates. Mechanisms ensuring the maintenance of cellular identity must be robust, as changes in cell fate appear to be fairly uncommon in wild-type conditions, with only one documented case of a regulated, complete fate switch being described in *Caenorhabditis elegans* (Jarriault et al., 2008). However, rare cases of transdifferentiation have been observed in mutants in which chromatin complexes are affected, suggesting a role for chromatin structure in the maintenance of cellular identity (Petrella et al., 2011; Tursun et al., 2011). A notable example is given by mutations affecting the *Drosophila lethal (3) malignant brain tumor* (*l(3)mbt*) gene, which cause malignant brain tumors that ectopically express germline-specific genes and have been proposed to be soma-to-germline transformations (Gateff et al., 1993; Janic et al., 2010). Gene expression profiling of *l(3)mbt* brain tumors and L(3)mbt-depleted cultured somatic cells identified a group of upregulated genes known as the malignant brain tumor signature (MBTS) that is enriched for factors specifically expressed in germ cells (Georlette et al., 2007; Janic et al., 2010; Meier et al., 2012; Sumiyoshi et al., 2016). Mutations of germline-specific genes, including those impairing the Piwi-interacting RNA (piRNA) factors *piwi*, *aub* and *vasa*, as well as the translational repressor *nanos*, were found to suppress the neural overgrowth induced by loss of L(3)mbt (Janic et al., 2010; Rossi et al., 2017). A subsequent study provided evidence that the Hippo growth control pathway is crucial for *l(3)mbt* mutant brain overgrowth, suggesting an alternative cause of tumorigenesis (Richter et al., 2011). Furthermore, our lab showed that strong *l(3)mbt* mutations cause a maternal, germline autonomous phenotype that precludes normal embryonic development, including primordial germ cell formation (Yohn et al., 2003). Together, these studies suggest that L(3)mbt could impart many functions in regulation of tissue identity.

*l(3)mbt* encodes a 1477 amino acid protein that is ubiquitously expressed in *Drosophila* and is conserved from worms to humans. L(3)mbt is thought to be a chromatin reader and harbors three MBT repeats that bind methylated histone tails *in vitro* as well as a zinc-finger domain (Bonasio et al., 2010). L(3)mbt is enriched at the promoters of repressed genes, suggesting a direct role in transcriptional repression, but its binding sites overlap with insulator elements, indicating that L(3)mbt might also function as an insulator accessory factor (Richter et al., 2011; Van Bortle et al., 2014). Notably, L(3)mbt was purified in two non-enzymatic repressive chromatin complexes: the *Drosophila* RBF, E2F2 and Myb-interacting proteins (dREAM complex, also called Myb-Muv B) as well as the L(3)mbt-interacting complex (LINT complex) (Lewis et al., 2004; Meier et al., 2012). dREAM is a multi-subunit complex that controls gene expression throughout the cell cycle but also represses developmental genes. L(3)mbt associates at sub-stoichiometric levels with dREAM and is strictly found in its repressive forms (Georlette et al., 2007; Lewis et al., 2004). The LINT complex is composed of L(3)mbt, the novel transcriptional repressor Lint-1 and the co-repressor CoREST, and has been shown to silence developmental genes in cultured cells (Meier et al., 2012). Interestingly, the dREAM and LINT complexes repress overlapping sets of genes in somatic cells, including genes that are normally expressed in the germline. Despite extensive biochemical studies, we still know little about which chromatin complex mediates L(3)mbt's role in tissue identity.

**Fig. 1.**
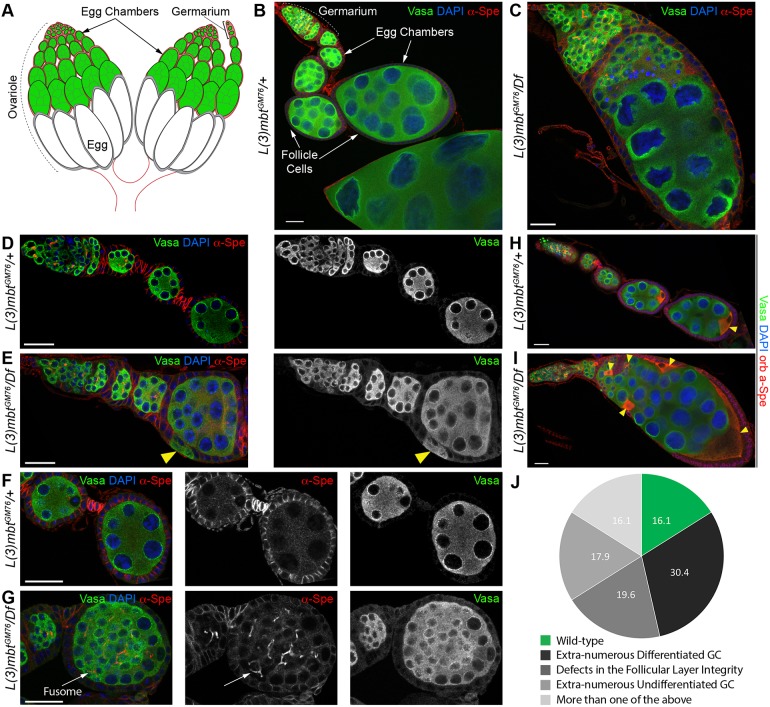
**Developmental defects of *l(3)mbt* mutant ovaries.** (A) Schematic of a wild-type ovary composed of ovarioles. (B-G) Confocal images of control and *l(3)mbt* mutant ovarioles stained for germ cells (Vasa, green), α-Spectrin (red), and with DAPI (blue) for DNA. All images are displayed with anterior oriented to the top-left corner. (B) Heterozygous control ovariole. (C) Representative *l(3)mbt* mutant ovariole with extra-numerous undifferentiated and differentiated germ cells surrounded by follicle cells. (D) Tip of wild-type ovariole with germarium and early egg chambers. (E) Mutant ovariole with defects in follicle cell layer integrity. Vasa-expressing germ cells appear intercalated between follicle cells (yellow arrowhead). (F) Wild-type stage 3 and 4 egg chambers. Egg chambers are separated by stalk cells (high spectrin signal) and germ cells within egg chamber are no longer connected by fusomes. (G) Similarly staged mutant egg chamber filled with fusome-containing undifferentiated germ cells (arrow). (H,I) Confocal images of control and mutant ovarioles stained for Vasa (green), Orb (oocyte marker), α-Spectrin (red) and with DAPI (blue). (H) In control ovarioles, Orb is restricted to the developing oocyte at the posterior of egg chambers. (I) *l(3)mbt* mutant ovariole with an egg chamber containing more than 16 germ cells and multiple oocytes, as revealed by Orb staining. Arrowheads in H,I indicate developing oocytes. (J) Quantification of phenotypes observed in *L(3)mbt* mutant ovarioles as illustrated in C,E,G,I), *n*=56. Scale bars: 25 μm.

*Drosophila melanogaster* ovaries are each composed of 16- to 20-egg assembly chains called ovarioles (Fig. 1A,B). At the tip of each ovariole a region called the germarium houses germline stem cells (GSCs), which divide asymmetrically to generate a new GSC and a differentiating daughter cell. The differentiating GSC daughter undergoes four rounds of mitosis with incomplete cytokinesis to form a 16-cell germline cyst in which sibling germ cells remain interconnected through cytoplasmic bridges called ring canals. GSCs are marked by a spectrin-containing spherical endoplasmic reticulum-derived vesicle known as a spectrosome, which fuses into a branched fusome connecting the cells of the same cysts through the ring canals (Huynh, 2006). Only one of the cyst germ cells develops into an oocyte; the other 15 cells become supportive, polyploid nurse cells. Somatic cells of the ovary play important roles in supporting oogenesis: they compose the GSC niche that promotes GSC divisions and cyst differentiation, and the follicle cells enclose and individualize egg chambers, being required for proper oocyte-nurse cell development.

To understand how L(3)mbt secures tissue identity, we combined genetic and genomic approaches to characterize the functions of L(3)mbt in *Drosophila* ovarian development. We find that the loss of L(3)mbt affects gene expression in a tissue-specific manner. In somatic cells of the ovary, L(3)mbt represses germline genes, whereas in the female germline it controls genes normally expressed in the testis and the nervous system. Mutant ovarian tissues continue to express signatures of the tissue of origin, indicating that loss of L(3)mbt does not induce transdifferentiation. Remarkably, we show that ectopic expression of a single gene in the somatic ovarian cells, the translational repressor and key regulator of germline fate *nanos*, is largely responsible for aberrant development. Using a genetic approach, we find that in the ovary L(3)mbt function requires its co-factor Lint-1 but is independent of the dREAM complex. Together, our experiments provide insight into the role of L(3)mbt in securing tissue identity by repressing expression signatures characteristic of other tissues that are incompatible with normal development.

## RESULTS

### Sterility in *l(3)mbt* mutant females is associated with aberrant ovarian development

L(3)mbt was previously shown to be required for the development of the nervous system as *l(3)mbt* mutant flies grown at restrictive temperatures (29°C) develop malignant brain tumors and die at larval stages (Gateff et al., 1993; Janic et al., 2010; Richter et al., 2011). In contrast, when grown at lower temperatures (18°C or 25°C), null *l(3)mbt* mutant females were viable but fully sterile, indicating that L(3)mbt is required for germline development independently of temperature. At the macroscopic level, *l(3)mbt* mutant ovaries were atrophied and adult females did not lay eggs. To characterize the ovarian phenotype in detail, we used antibodies against the germline marker Vasa and α Spectrin (α-Spe), which labels the membranes of somatic cells and spectrosomes/fusomes. Similar to the wild type, *l(3)mbt* mutant germaria contained GSCs adjacent to the somatic niche (Fig. S1A,B). However, mutant ovarioles contained fewer individualized egg chambers (1.35 egg chambers/ovariole on average versus 6 in wild type). *l(3)mbt* mutant egg chambers occasionally contained apoptotic cells, were highly abnormal and displayed several defects (Fig. 1C,E,G, quantified in 1J). Such defects included extra-numerous germ/nurse cells within egg chambers (Fig. 1C; ‘extra-numerous differentiated GC’ in Fig. 1J) as well as egg chambers in which the layer of somatic follicle cells does not fully enclose germ cells (Fig. 1E, arrowhead; ‘defects in the follicular layer integrity’ in Fig. 1J). In the wild type, fusomes degenerate as cysts proceed past the 16-cell stage and are enveloped by somatic follicle cells (Huynh and St Johnston, 2004). In *l(3)mbt* mutants, however, we observed accumulation of undifferentiated germ cells, which were marked by branched fusomes and enclosed by follicle cells (Fig. 1G, arrow; ‘extra-numerous undifferentiated GC’ in Fig. 1J).

Another hallmark of the 16-cell wild-type cyst is the specification of a single oocyte, while the remaining 15 cells develop into polypoid feeder cells, the nurse cells. Using the oo18 RNA-binding protein (Orb; Christerson and McKearin, 1994) as a marker for the future oocyte (Fig. 1H, arrowhead), we observed that most mutant egg chambers had multiple Orb-positive cells (Fig. 1I, arrowheads). Wild-type oocytes are connected to the adjacent nurse cells by four ring canals, as a product of four divisions (Huynh and St Johnston, 2004). We determined whether the additional cells were bona fide oocytes by counting the associated ring canals (stained by F-Actin; Fig. S1C,D, yellow arrows and dashed circles) and found that ectopic Orb-expressing cells contained four or more ring canals in egg chambers with multiple oocytes and extra-numerous germ cells. Thus, *l(3)mbt* loss causes egg chamber fusions and, possibly, additional rounds of cyst division. In 2% of the examined ovaries (*n*>200), we observed multiple germaria connected to the same aberrant egg chamber (Fig. S1E,F), suggesting that ovarioles were fused during ovary morphogenesis. Taken together, our results indicate that, in addition to its previously reported, conditional requirement in the brain, L(3)mbt has an essential, temperature-independent role required for ovarian morphogenesis and differentiation.

### L(3)mbt functions in somatic ovarian cells to safeguard ovary development

**Fig. 2.**
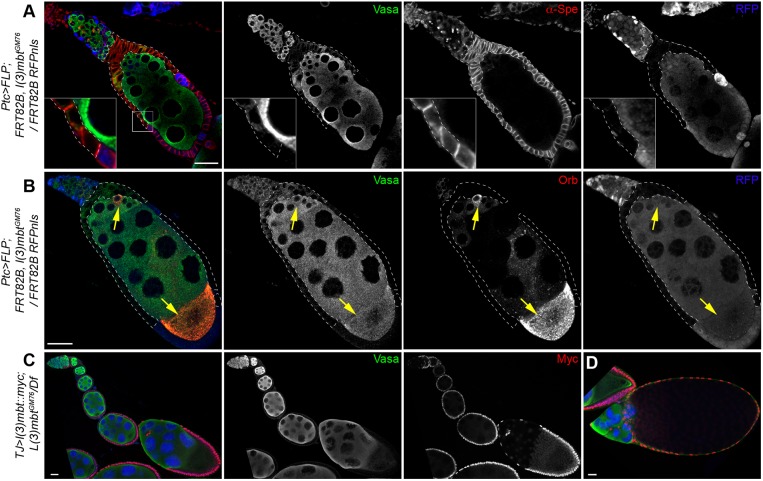
**L(3)mbt functions in somatic cells for ovary development.** (A,B) Confocal images of representative ovarioles with mutant *l(3)mbt* follicle cell clones marked by absence of RFP (blue), Vasa (green), α-Spectrin (A) or Orb (B) (red); mutant clones are outlined by dashed lines. (A) An egg chamber surrounded by numerous *l(3)mbt* mutant follicle cells exhibits *l(3)mbt* characteristic phenotypes such as supernumerary germ cells and disrupted follicle cell epithelial layer. Insets show magnified view of the boxed area. (B) An egg chamber surrounded by *l(3)mbt*-depleted follicle cells contains more than one oocyte (yellow arrows). (C,D) Confocal images of mutant ovaries expressing *TJ>UAS-l(3)mbt::myc* in somatic cells stained for Vasa (green), Myc (red), and with DAPI (blue). (C) 94.5% of mutant ovarioles expressing the L(3)mbt wild-type transgene in somatic cells have normal morphologies and germ cell numbers (*n*=201). (D) Rescued late-stage oocyte. Scale bars: 25 μm.

Previous experiments had shown that loss of *l(3)mbt* specifically in germ cells caused developmental defects in the resulting embryos, but allowed oocytes to mature (Yohn et al., 2003). Thus, we wondered whether the gross abnormalities of mutant ovaries were indicative of a role for L(3)mbt in somatic cells of the ovary. To determine the tissue-specific requirement of L(3)mbt, we generated homozygous *l(3)mbt^GM76^* mutant clones in the somatic ovarian cells by using the FRT-FLP system (Harrison and Perrimon, 1993) under the transcriptional control of the *ptc-Gal4* driver, which drives expression in the somatic cells of the germarium (Hinz et al., 1994)*.* Interestingly, loss of L(3)mbt in multiple somatic cells perturbed germline development, leading to egg chambers that contained extra-numerous germ cells (Fig. 2A) or multiple oocytes (Fig. 2B). To test conclusively for a role of L(3)mbt in somatic ovarian cells, we expressed an inducible *UAS-l(3)mbt::myc* transgene under the control of the *tj-Gal4* driver, which is specifically expressed in the somatic cells of the ovary. We found that expression of L(3)mbt in the somatic cells of the ovary alone was sufficient to rescue the aberrant morphology of mutant ovaries, including number of oocytes and ring canal (Fig. 2C,D, Fig. S2A,B). This rescue was highly penetrant with only 5.5% of somatically complemented egg chambers exhibiting the phenotypes shown in Fig. 1 (*n*=201). These results demonstrate that L(3)mbt is required specifically in the somatic tissues of the ovary to support normal oogenesis.

### Mutant larval somatic cells are properly specified but intermingled cells fail to contact germ cells

**Fig. 3.**
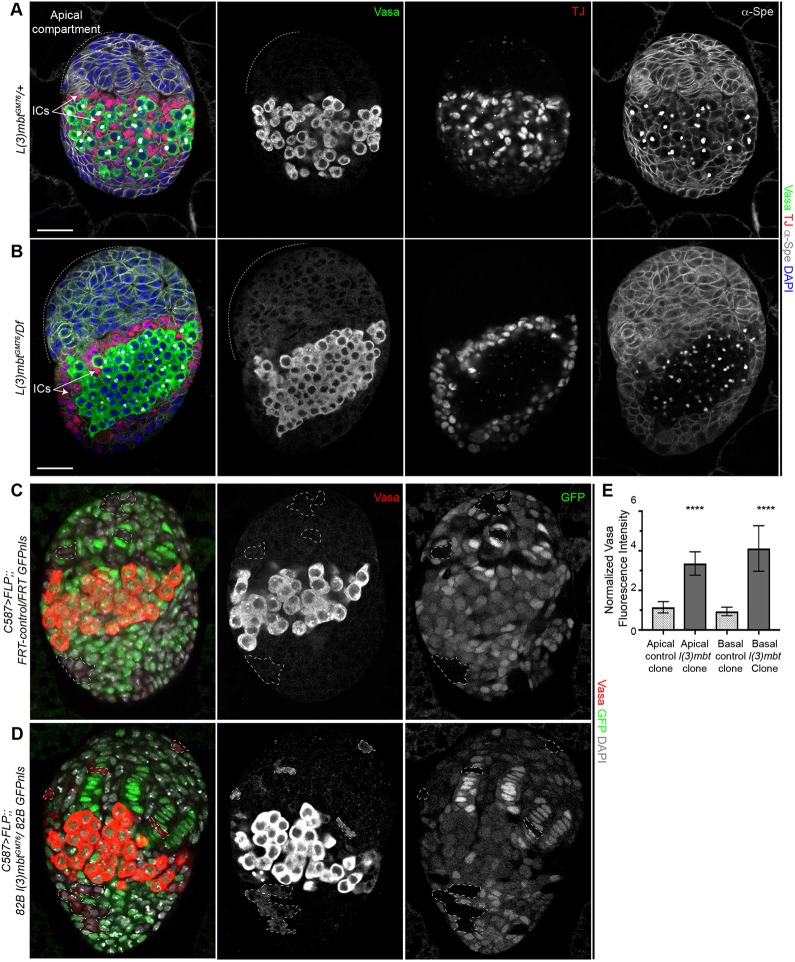
**Larval somatic cells are properly specified but de-repress Vasa.** (A,B) Wild-type (A) and *l(3)mbt* mutant (B) L3 ovaries stained for Vasa (green), TJ (red), α-Spectrin (gray) and with DAPI (blue). *l(3)mbt* mutant ICs fail to migrate in between PGCs and are instead surrounding them. (C,D) Confocal images of L3 ovaries with wild-type (C) or *l(3)mbt* mutant clones (D) marked by the absence of GFP. Ovaries stained for Vasa (red), GFP (green) and with DAPI (gray). *l(3)mbt^GM76^* mutant clones in somatic cells express Vasa whereas wild-type clones do not. (Selected clones are outlined by dashed lines.) (E) Quantification of the normalized Vasa fluorescence intensity in control and *l(3)mbt* mutant clones. ****P<10^−4^, unpaired *t*-test. Scale bars: 25 µm.

Ovarioles lacking L(3)mbt exhibit striking morphological defects. Most of the structures in the adult ovary are established and organized during the third instar larval (L3) stage (Gilboa, 2015); we thus examined ovaries from mid to late L3 larvae to investigate whether *l(3)mbt* mutation affects ovarian organogenesis. Germ cells and somatic cells associate during late embryonic stages and proliferate during most of larval development. Starting at the mid L3 stage, the somatic precursors differentiate into distinct populations of somatic cells (Gilboa and Lehmann, 2006; Li et al., 2003). In the apical compartment, post-mitotic terminal filament cells stack to form terminal filaments and associate with sheath cells (Godt and Laski, 1995). In the medial region, the intermingled cells (ICs) are closely associated with germ cells and are thought to give rise to the adult escort cells (Gilboa, 2015). We performed confocal imaging analysis by immunostaining with antibodies against the transcription factor Traffic Jam (TJ), which has important functions in specifying somatic gonadal cell types and labels the ICs (Li et al., 2003), Vasa and α-Spec. We observed that, like their wild-type counterparts, *l(3)mbt* mutant L3 ovaries have distinct apical compartments harboring terminal filaments, as well as a medial region containing germ cells and ICs (Fig. 3A,B). ICs are normally scattered throughout the germ cell population and an arbitrarily defined volume centered on the germ cell compartment contained an average of 216 ICs in control ovaries (Fig. S3A). However, mutant ICs were excluded from the germ cell-containing region and we only observed 24 somatic cells in a similarly sized germ cell containing region (Fig. 3B, Fig. S3A). Despite this aberrant behavior, *l(3)mbt* mutant ICs retained expression of Zfh1, a transcription factor essential for the somatic fate (Fig. S3B) (Maimon et al., 2014). Taken together, our results show that in *l(3)mbt* mutant L3 ovaries, markers for somatic cell fates are expressed but spatial organization is affected.

### *l(3)mbt* mutant somatic larval cells ectopically express the germline marker Vasa

Previous studies suggested that L(3)mbt loss results in de-repression of germline genes in the larval brain and cultured somatic cells (Georlette et al., 2007; Janic et al., 2010; Meier et al., 2012; Sumiyoshi et al., 2016). Thus, we investigated whether germline genes were ectopically expressed in mutant larval somatic cells. We observed faint Vasa antibody signal in the somatic tissues of *l(3)mbt* mutant ovaries, especially in the apical compartment (Fig. 3B, Fig. S3). To extend these initial observations, we induced *l(3)mbt* mutant clones using the *c587-Gal4* driver to express FLP recombinase specifically in the somatic tissues of the larval ovary (Zhu and Xie, 2003). Induction of wild-type control clones (identified by the absence of GFP) in somatic cells of the basal or apical compartment did not cause Vasa expression (Fig. 3C,E). In contrast, *l(3)mbt^GM76^* homozygous clones of somatic cells exhibited Vasa staining (Fig. 3D, quantified in 3E). Of note, we also observed *vasa* derepression in *l(3)mbt* mutant clones in adult somatic cells (Fig. 2A, inset). From this, we conclude that *L(3)mbt* represses the germ cell marker *vasa* in the somatic cells of the larval ovary.

### *l(3)mbt* mutant somatic ovarian cells simultaneously express somatic gonad and germline-specific genes

**Fig. 4.**
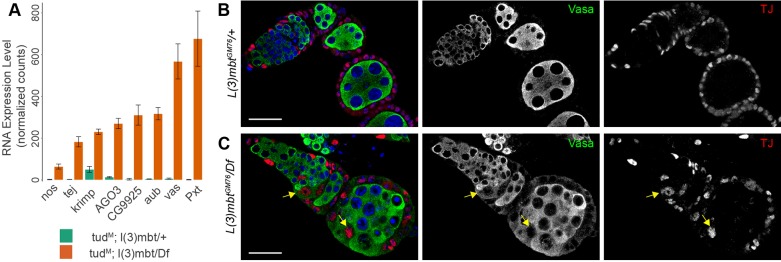
***l(3)mbt* mutant somatic cells are properly specified but ectopically express germline genes.** (A) RNA expression analysis of *tud^M^* ovaries, which lack germ cells, heterozygous and homozygous mutant for *l(3)mbt*. Expression level of the germline-specific genes *nos*, *tej*, *krimp*, *AGO3*, *CG9925*, *aub*, *vas* and *Pxt* as measured by RNA-seq analysis (expressed in normalized counts). (B,C) Confocal images of control and *l(3)mbt* mutant ovarioles stained for Vasa (green), Traffic Jam (TJ; red) and with DAPI (blue). TJ is expressed in all somatic cells of the adult ovary. (C) Some TJ-positive somatic cells express the germline marker Vasa (yellow arrows). Scale bars: 25 μm.

To gain a genome-wide view of gene expression changes induced by loss of L(3)mbt in adult somatic ovarian cells *in vivo*, we performed RNA-sequencing (RNA-seq) analysis. To distinguish between germline and somatic ovarian tissues, we took advantage of *tud* maternal mutations (*tud^M^*), which give rise to progeny that lack germ cells and develop into adults devoid of germline (Arkov et al., 2006; Smendziuk et al., 2015; see the supplementary Materials and Methods and the Materials and Methods). Comparisons between *tud^M^; l(3)mbt^GM76^/l(3)mbt^Df^* and *tud^M^; l(3)mbt^GM76^/+* adult ovaries identified 600 differentially expressed genes (adjusted *P*-value<0.05) in the somatic cells of the ovary. Of these, 459 were upregulated and 141 downregulated in mutant tissues (Table S1). Forty-four upregulated genes are shared with the 101 MBTS genes identified in *l(3)mbt* tumorous brains (Janic et al., 2010), and 116 upregulated genes were also among the 681 genes found to be upregulated in *Δ*−*l(3)mbt* ovarian somatic cells (OSCs; Sumiyoshi et al., 2016). These de-repressed genes include piRNA pathway components and germline-specific genes such as *nos*, *Pxt*, *vas*, *aub*, *tej*, *krimp*, *AGO3* and *CG9925* (Fig. 4A, Fig. S4A). The effect of *l(3)mbt* mutation on gene expression was more pronounced for repressed genes, whereas genes normally expressed in the soma showed only low fold-changes in the mutant (124/141 had a log_2_ fold-change between −0.3 and −1). To investigate whether mutant somatic cells retained their somatic identity, we performed immunohistochemistry for the key transcription factors Traffic Jam (TJ) and Zfh1, which are essential for gonad development and are exclusively expressed in somatic cells (Leatherman and Dinardo, 2008; Li et al., 2003). Despite the gross morphological abnormalities, we observed TJ- or Zfh1-expressing cells surrounding germ cells in mutant ovaries (Fig. 4B,C, Fig. S4B,C). Furthermore, F-Actin staining showed that mutant somatic cells retain the columnar morphology and epithelial characteristics of wild-type follicle cells (Fig. S1D, Fig. S2A). However, we detected TJ-positive cells that simultaneously expressed the germline marker Vasa in 36% of mutant egg chambers (Fig. 4C, arrows; *n*=100). As TJ/Zfh1 and Vasa expressions are mutually exclusive in wild-type ovaries, our results indicate that *l(3)mbt* mutant somatic cells retain somatic and epithelial/follicular characteristics while ectopically expressing hallmark genes of germline fate.

### Expression of Nos is necessary and sufficient to cause egg chamber fusion

**Fig. 5.**
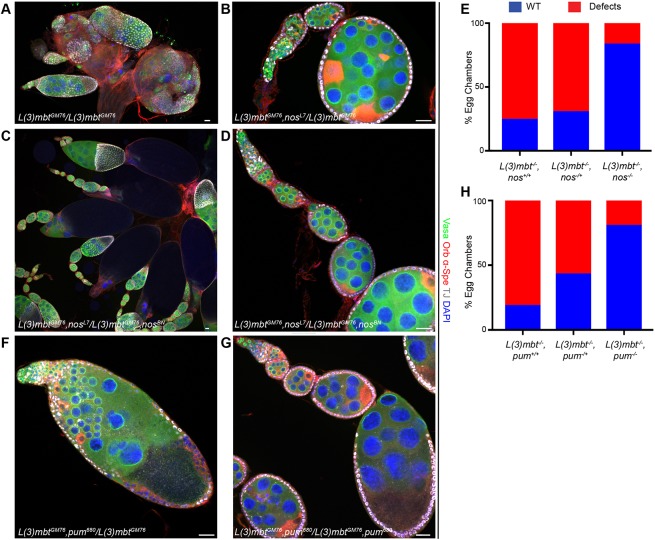
**Nanos and its co-factor Pumilio mediate developmental phenotypes in *l(3)mbt* ovaries.** (A-D,F,G) Confocal images of ovaries stained for Vasa (green), Orb and α-Spectrin (red), TJ (gray) and with DAPI (blue). (A-D) Representative confocal images of (A) *l(3)mbt^GM76^*, (B) *l(3)mbt^GM76^, nos^L7^/l(3)mbt^GM76^, +* and (C,D) *l(3)mbt^GM76^,* *nos^L7^/l(3)mbt^GM76^,* *nos^BN^* double mutant ovarioles. (E) Quantification of phenotypes (combined as described in Fig. 1) observed in the genotypes described in A-D. (F,G) Confocal images of (F) *l(3)mbt^GM76^,* *pum^680^*/*l(3)mbt^GM76^,* + and (G) *l(3)mbt^GM76^,* *pum^680^* homozygous ovarioles. (H) Quantification of phenotypes observed in the genotypes described in F,G (*nos, l(3)mbt: n*=88; *pum, l(3)mbt*: *n*=181). Scale bars: 25 μm.

**Fig. 6.**
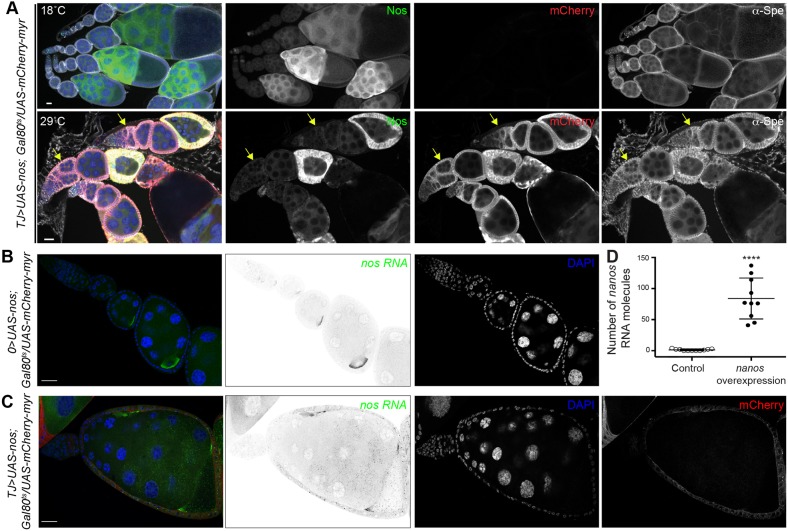
***Nanos* ectopic expression in somatic cells causes egg chamber fusion.** (A) Confocal images of ovaries expressing *UAS-nos; UAS-myr-mCherry* transgenes in somatic cells using a temperature-sensitive system (Gal80^ts^) to express *nos* only in the adult. Ovaries were stained for Nos (green), mCherry (red), α-Spectrin (gray) and with DAPI (blue). At 18°C, the transgenes are not expressed and ovarioles develop normally. When shifted to 29°C after eclosion, ectopic *nos* expression in somatic cells perturbs egg chamber individualization (arrows). Note variable expression of Nos protein in somatic cells. (B,C) Representative images of *nos* smRNA FISH in ovarioles; *nos* RNA is shown in green and grayscale, mCherry in red, and nuclei are stained with DAPI (blue). (B) In control ovarioles (*0*>, no driver), *nos* transcripts accumulate in nurse cells and oocytes, but not in somatic cells. (C) At 29°C, ovarioles expressing *UAS-nos; UAS-mCherry-myr* in somatic cells exhibit egg chamber fusion. (D) Quantification of absolute number of *nos* RNA molecules in control and *UAS-nos; UAS-myr-mCherry*-expressing cells (*n*=10). ****P<10^−4^, unpaired *t*-test. Scale bars: 25 µm.

Aberrant growth of *l(3)mbt* brain tumors has been shown to rely on the ectopic expression of *nos*, *aub* and *vasa* (Janic et al., 2010). We find that each of these genes is indeed upregulated in compromised somatic ovarian cells lacking L(3)mbt (Fig. 4A, Fig. S4A). We therefore investigated whether their misexpression contributed to the ovarian defects of *l(3)mbt* mutants by generating double mutant animals. Owing to lethality, we were unable to assess ovarian phenotypes in *vas, l(3)mbt* mutants. However, we found that *aub, l(3)mbt* double mutant ovaries were phenotypically similar to the *l(3)mbt* single mutant, containing many apoptotic cells and aberrant egg chambers with more than 16 germ cells (Fig. S5A). In contrast, *nos^BN^/nos^L7^* mutations dramatically suppressed the *l(3)mbt* ovarian defects: *nos, l(3)mbt* double mutant ovaries contained late-stage egg chambers with ovarioles that were phenotypically indistinguishable from wild type, and 85% of double mutant egg chambers contained 16 germ cells and only one oocyte (Fig. 5A-E; *n*=88). Consistent with this, depletion of Nanos' co-factor Pumilio in *l(3)mbt* mutant ovaries also suppressed the mutant phenotypes, with 81% of *pum*^680^*,* *l(3)mbt* double mutant ovarioles resembling wild-type morphology (Fig. 5F-H; *n*=181). These data suggest that Nanos and Pum are crucial factors leading to *l(3)mbt* mutant ovarian phenotypes. To test whether *nos* misexpression in somatic ovarian cells is sufficient to cause *l(3)mbt*-like ovarian defects, we ectopically expressed *nos* in the somatic cells of the ovary using the *tj-Gal4* driver. *nos* misexpression during larval stages caused lethality; we therefore restricted *nos* expression in the soma to adult stages using the Gal80^ts^ system (McGuire et al., 2004). In these conditions, *nos* somatic expression perturbed ovarian morphology: 47% of ovarioles examined contained at least one egg chamber with extra-numerous germ cells and/or oocytes, reminiscent of the egg chamber fusions observed in *l(3)mbt* mutants (*n*=116; Fig. 6A-C). We quantified *nos* overexpression in follicle cells using single molecule RNA fluorescence *in situ* hybridization (FISH) (Trcek et al., 2015, 2017). In control follicle cells, we detected an average of 1.27 *nos* RNA molecules (Fig. 6B, quantified in 6D). In contrast, *UAS-nos, UAS-mCherry*-expressing follicle cells contained on average 84 *nos* RNA molecules (Fig. 6C,D, Fig. S6A,B). In comparison, a similarly sized region of interest (ROI) of the oocyte contained 110 *nos* transcripts. In contrast to *nos*, ectopic expression of *aub* or *vas* in somatic ovarian cells did not yield a morphologically significant phenotype (Fig. S6C,D). Together, these results suggest that ectopic expression of *nos*, but not *aub *or* vas*, is necessary and sufficient to cause aberrant somatic ovarian development.

### L(3)mbt functions through the LINT complex to secure ovarian development

**Fig. 7.**
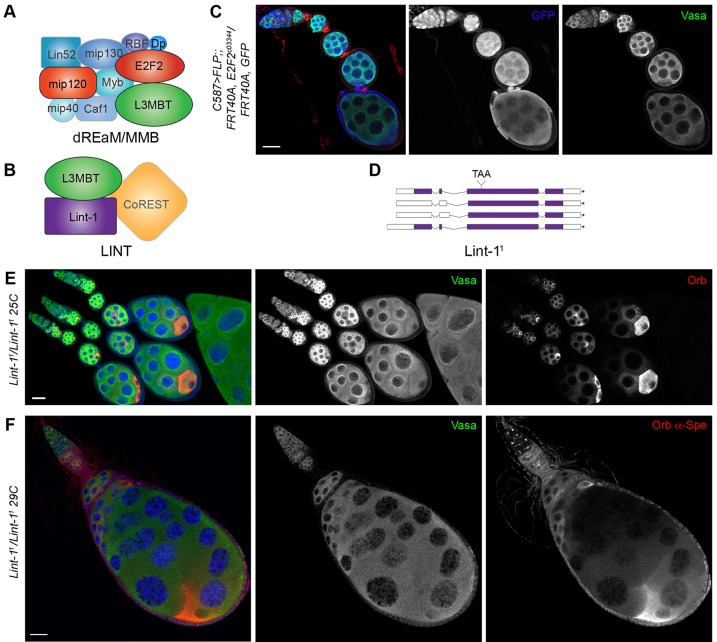
**LINT complex mutants have ovarian defects similar to *l(3)mbt*.** (A,B) Schematic of the dREAM/MMB (Georlette et al., 2007) (A) and LINT (B) complexes (Meier et al., 2012). (C) Confocal image of ovariole with *E2f2^c03344^* mutant clones marked by absence of GFP (blue), Vasa (green), α-Spectrin (red). (D) Schematic of the *Lint-1^1^* allele. (E,F) Confocal images of *Lint-1* mutant ovaries stained for Vasa (green), Orb (red), with DAPI (blue) and for α-Spectrin (red in F). (E) At 25°C, 2% of *Lint-1^1^* mutant egg chambers contain two Orb-positive cells or mis-positioned oocytes (*n*=100). (F) At 29°C, 15% of *Lint-1^1^* ovarioles contain egg chambers with more than 16 germ cells and multiple oocytes similar to defects observed in *l(3)mbt* mutants (*n*=100). Scale bars: 25 μm.

L(3)mbt has been associated with two chromatin complexes that repress developmental and germline genes: the dREAM and LINT complexes (Fig. 7A,B; Georlette et al., 2007; Meier et al., 2012). To determine the function of these complexes in ovarian development, we depleted somatic cells of E2F2, the major repressor of the dREAM complex. Mutant *E2f2*-depleted somatic clones did not exhibit phenotypic aberrations reminiscent of those observed in *l(3)mbt* mutants (Fig. 7C). Similarly, ovaries deficient for Mip120, another repressor and core member of the complex, did not recapitulate the somatic phenotypes found in *l(3)mbt* mutants (Fig. S7). Together, these results suggest that L(3)mbt's crucial function in somatic ovarian cells is independent of the dREAM complex.

Next, we examined the role of the LINT complex as a mediator of L(3)mbt function. Since mutations in *Lint-1* had not been identified, we generated CRISPR-induced *Lint-1* alleles (Gratz et al., 2013, 2014). *Lint-1^1^* deletes two cytosines (350 and 351), creating a premature stop codon at position 66/540 (Lint-1-C) or 128/601 (Lint-1-A; Fig. 7D). Homozygous mutant flies were viable and fertile at 25°C, and their ovaries developed normally although 2% of egg chambers contained misplaced or extra-numerous oocytes (Fig. 7E; *n*=100). However, when grown at 29°C, *Lint-1^1^* females were fully sterile, laying eggs that failed to hatch, and 15% of their ovarioles developed aberrantly and contained extra-numerous germ cells and oocytes similar to the egg chamber fusions caused by *l(3)mbt* mutation (Fig. 7F; *n*=100). Furthermore, depletion of one *l(3)mbt* copy rendered *Lint-1^1^* homozygous females (*Lint-1^1^/ Lint-1^1^;;l(3)mbt^GM76^/+*) fully sterile at 25°C, strongly suggesting a genetic interaction. We conclude that L(3)mbt's function in the ovary is mediated by its co-factor Lint-1 and the LINT complex.

### L(3)mbt is autonomously required in the female germline for egg chamber survival and to repress neuronal and testis-specific genes

**Fig. 8.**
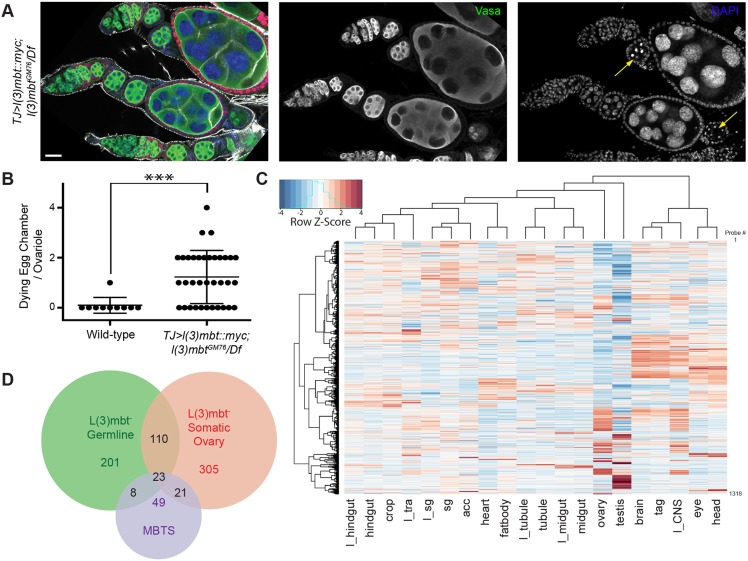
**L(3)mbt represses neuronal and testis-specific genes in the germline.** (A) Confocal image of *l(3)mbt* mutant ovarioles expressing the *l(3)mbt::myc* transgene in somatic cells, stained for Vasa (green), Myc (red), F-Actin (gray) and with DAPI (blue). Scale bar: 25 μm. Some *l(3)mbt* mutant egg chambers surrounded by somatic cells expressing the L(3)mbt::myc fusion undergo cell death (yellow arrows). (B) Quantification of egg chambers undergoing cell death. *n*=35; ***P<10^−3^, unpaired *t*-test. (C) Hierarchical clustering of the tissue expression profiles of genes repressed by L(3)mbt in the female germline. Gene expression per tissue (normalized to fly average) is shown as a *z*-score heatmap. (D) Venn diagram showing genes upregulated in *l(3)mbt* mutant ovarian soma (red), female germline (green), and larval brain tumors (MBTS) (Janic et al., 2010). Most de-repressed genes are tissue specific.

In addition to its role in the development of the somatic cells of the *Drosophila* ovary, L(3)mbt also has a maternal, germline-autonomous role that supports nuclear divisions during early embryogenesis (Yohn et al., 2003). Similar to previous results obtained with mutant germline clones, we observed that embryos laid by mutant females, which somatically expressed the complementing *l(3)mbt::myc* transgene, failed to hatch. Furthermore, although most egg chambers appeared morphologically normal in somatically complemented *l(3)mbt* mutant ovaries, we noticed that 69% of ovarioles contained at least one apoptotic egg chamber (Fig. 8A,B; *n*=35). This suggests that, in addition to its previously identified maternal effect function for early embryonic development, L(3)mbt is autonomously required in the germline for egg chamber development.

Our data highlight a role for L(3)mbt in suppressing germline-specific genes in somatic tissues of the ovary. However, our experiments also uncovered a germline autonomous requirement for egg chamber development. To gain a genome-wide view of the changes in gene expression specifically induced by loss of L(3)mbt in the germline, we performed RNA-seq analysis on embryos laid by *l(3)mbt* mutant or heterozygous mothers expressing the *L(3)mbt::myc* fusion in the somatic ovary. We used early embryos prior to activation of the zygotic genome as the early embryonic RNA pool is exclusively composed of maternally provided transcripts and thereby reflects the germline expression profile during oogenesis (Edgar and Schubiger, 1986). Our analysis identified 878 differentially expressed genes (adjusted *P*-value<0.05), of which 342 were upregulated and 536 downregulated in embryos laid by mutant females (Table S2). Most upregulated genes were uncharacterized and not enriched for specific gene ontology terms. Thus, to better characterize this group of genes upregulated in the *l(3)mbt* mutant germline, we performed two-way hierarchical clustering to identify any tissue-specific expression signatures. Two major groups were readily identified: one composed of genes highly expressed in neuronal tissues [brain, thoracicoabdominal ganglion (tag), larval CNS, eye, and head; Fig. 8C] and another comprising testis-specific genes. As in mutant somatic ovaries, most downregulated genes had low fold changes compared with the heterozygous controls (365/536 had a log_2_ fold change between −0.46 and −1; Fig. S8). We observed only a limited overlap between the genes that were de-repressed in *l(3)mbt* mutant somatic and germline ovarian tissues, with two-thirds of genes being specifically upregulated in one of the two tissues (Fig. 8D). Taken together, these results indicate that L(3)mbt function is not restricted to repressing the germline program in somatic tissues, but that L(3)mbt regulates distinct sets of genes in a tissue-specific manner.

## DISCUSSION

By combining developmental and molecular analysis, we show that *l(3)mbt* mutant ovaries develop aberrantly. L(3)mbt depletion does not result in complete transdifferentiation but causes simultaneous expression of original cell signatures and ectopic expression of markers of other cell fates. We hypothesize that the conflict between co-existing cell identities causes the observed aberrant tissue morphogenesis. Direct support for this idea is provided by the role of the translational repressor and germline gene *nanos*, de-repression of which in the somatic cells causes aberrant growth. Molecularly, we demonstrate that L(3)mbt functions, at least in part, through the LINT complex in the somatic ovary. Finally, we show that L(3)mbt-mediated regulation of gene expression is not limited to repression of germline-specific genes in somatic tissues but is tissue dependent. We propose that L(3)mbt functions, through LINT, as a guardian of cell identity by preventing the simultaneous expression of gene sets incompatible with such identity.

Our experiments demonstrate that ectopic expression of *nanos* is necessary and sufficient to induce aberrant development of *l(3)mbt* mutant ovaries. These defects are unlikely to be due to a direct interference at the transcriptional level but are rather caused by Nos' function as a translational repressor. In support of this, we find that Pumilio, a sequence-specific translational repressor and co-factor of Nos, is also essential for the *l(3)mbt* ovarian phenotype. Nos was recently shown to modulate Pum RNA binding and target specificity in somatic S2 cells (Weidmann et al., 2016). Since Pum is ubiquitously expressed, we propose that ectopic Nos stabilizes Pum binding at target mRNAs essential for somatic functions. Interestingly, ectopic expression of NANOS1 was found to be required for growth of human retinoblastoma tumor suppressor-deficient tumor cells. In this case, NANOS1 and PUM repress p53 translation allowing cells to bypass apoptosis (Miles et al., 2014). Thus, ectopic Nos-Pum complexes may alter tissue maintenance at the post-transcriptional level in other systems as well. As Nos has been found to repress somatic genes in germ cells of multiple organisms (Hayashi et al., 2004; Lee et al., 2017), we would expect to find key regulators of somatic fate among mRNAs aberrantly targeted by Nos-Pum in somatic *Drosophila* tissues.

In contrast to the widespread effects of *nos* de-repression observed in multiple somatic tissues upon loss of L(3)mbt, ectopic expression or activity of additional genes may define the exact phenotypic consequences, which depend on tissue type (Rossi et al., 2017). For example, piRNA pathway genes are ectopically expressed in *l(3)mbt* larval brain tumors and somatic ovarian cells; however, depleting them ameliorates the brain tumor but not the ovarian phenotype. This difference could be explained by the fact that the somatic ovary already uses core components of the piRNA pathway to regulate transposable elements (Handler et al., 2013). Similarly, we did not observe de-repression of Hippo target genes in ovarian tissues. Consistent with our finding that in *l(3)mbt* mutants new and original tissue identities are co-expressed, these results suggest that the phenotypic consequences of *l(3)mbt* mutation depend on the context of the original tissue identity.

Our results demonstrate that L(3)mbt function in the ovary is independent of the dREAM complex. In salivary glands, the core repressor Mip120 is required to localize L(3)mbt to the chromosomes (Blanchard et al., 2014). However, in ovarian cells, L(3)mbt cytolocalization did not appear to be affected by Mip120 loss (Fig. S7). The dREAM complex has a well-established role in cell cycle regulation (Sadasivam and Decaprio, 2013). Indeed, Mip120, was recently found to be required for decondensation of nurse cell nuclei (Cheng et al., 2017) and E2F2 is required for endo-replication of follicle cells (Cayirlioglu et al., 2001). We did not observe a role for L(3)mbt in the regulation of nurse and follicle cell endo-replication. Instead, our data support the hypothesis that, in the ovary, L(3)mbt functions predominantly through the LINT complex and that this complex can be functionally separated from the dREAM complex. Considering the moderate phenotype of *Lint-1^1^* mutants and that *Lint-1* gene function is apparently dispensable at 25°C, we speculate that L(3)mbt exerts most of the repressive activity of this new complex, possibly with additional, as yet unidentified interactors, and that Lint-1 has an accessory role.

Loss of L(3)mbt causes the ectopic expression of a number of genes including cell identity regulators that interfere with original cellular function and affect tissue development. In contrast to a previously suggested soma-to-germline transformation, our results favor the hypothesis that *l(3)mbt* mutation imbalances tissue homeostasis whereby normally mutually exclusive lineage determinants become simultaneously expressed. In support of this, L(3)mbt depletion in neuronal, somatic ovarian, and germ cells does not lead to loss of original tissue-specific markers, but genes characteristic of other lineages are de-repressed (Richter et al., 2011; this study). Moreover, our results suggest that the role of L(3)mbt is not solely restricted to prevention of ectopic expression of germline genes, but instead L(3)mbt represses distinct, broader sets of genes in a tissue-specific manner. Therefore, we propose that L(3)mbt secures tissue identity by stabilizing the gene expression profiles established during differentiation.

## MATERIALS AND METHODS

### Fly stocks

*FRT82B, l(3)mbt^GM76^, e/TM6b* was generated in the R.L. lab (Yohn et al., 2003) and secondary mutations removed (Richter et al., 2011). The following stocks were obtained from the Kyoto Stock Center: *w^1118^;; Df(3R)ED19066/TM6c* (#150208) and y* w*; P{GawB}NP1624/CyO, P{UAS-lacZ.UW14}UW14 (tj-Gal4, #104055); from Bloomington Drosophila Stock Center: *w1118; P{neoFRT}82B P{Ubi-GFP(S65T)nls}3R/TM6B, Tb^1^* (BDSC #32655), y^1^ w*; P{UAS-FLP.D}JD1 (BDSC #4539), and w*; P{tubP-GAL80ts}2/TM2 (BDSC #7017). *C587-Gal4, UAS-nos-tub* (Clark et al., 2002; Ye et al., 2004), *UAS-vas* (Sengoku et al., 2006), *UASp-mCherry-myr, UAS-Aub-GFP* (Harris and Macdonald, 2001), and *TdTomato::l(3)mbt* (Blanchard et al., 2014) transgenes were obtained from the Xie, Jan, Nakamura, Zallen, Macdonald, and Botchan labs, respectively. The *mip120^67.9A.9^* (Beall et al., 2007) and *FRT40A e2f2c^03344^* (Ambrus et al., 2007) mutations were generated by the Botchan and Frolov labs, respectively. The following mutations are from R.L. lab stocks: *tud^1^, tud^B42^* (Arkov et al., 2006), *aub^HN2^, aub^QC42^* (Schupbach and Wieschaus, 1991), *nos^L7^* (Wang and Lehmann, 1991), *nos^BN^* (Wang et al., 1994) and *pum^680^* (Lehmann and Nüsslein-Volhard, 1987). All stocks were maintained at 18°C and crosses were performed at 25°C unless otherwise stated. The molecular nature of alleles is described in the supplementary Materials and Methods.

### Generation of transgenic lines

To generate *UASp-l(3)mbt::*myc transgenic flies*, l(3)mbt* coding sequence was amplified from LD05287 gold cDNA (*Drosophila* Genomics Resource Center), cloned using the p-ENTR/D-TOPO system and recombined into the pPWM destination vector (*Drosophila* Gateway Vector Collection) using Gateway technology (Invitrogen). *pPWM-l(3)mbt* was randomly inserted on the second chromosome through P-transposition. To generate the *Lint-1^1^* mutation, the target sequence (chrX:11044844-11044866) was identified by the flyCRISPR Optimal Target Finder tool (Gratz et al., 2014), amplified from genomic DNA using *lint-CRISPR* oligos (Table S3) and ligated in the pU6-*Bbs*I-gRNA plasmid (Gratz et al., 2013). The resulting construct was injected into *FRT19A;; vas-Cas9* embryos and progeny was screened by PCR and sequencing.

### Immunofluorescence

Adult ovaries from 2- to 3-day-old fattened females were dissected in cold PBS and fixed in 4% paraformaldehyde (PFA) for 20 min. Ovaries were permeabilized with 0.2% Triton X-100 in PBS and blocked with 1% (w/v) bovine serum albumin and 0.2% Triton X-100 (PBST). Samples were incubated with primary antibodies in PBST overnight at 4°C. Next, ovaries were washed and incubated with secondary antibodies in PBST for 2 h at room temperature. After three washes in PBS-0.2% Triton for 20 min each (including one containing 1:1000 DAPI), ovaries were mounted in SlowFade Gold mountant (Invitrogen) and imaged on Zeiss LSM780 or 800 confocal microscopes using 10×, 20× or 43× objectives. Larval ovaries were processed the same way but permeabilized for 4-8 h prior to primary antibody incubation. The following primary antibodies were used: rabbit anti-Vasa [1:5000, Gilboa and Lehmann (2004), Figs 1-8, Figs S1, S2, S5 and S6]; goat anti-Vasa (1:200, Santa Cruz Biotechnology sc-26877, Fig. S3); chicken anti-Vasa [1:200, Gilboa and Lehmann (2004), Fig. S4]; mouse anti-Spectrin (1:200, DSHB); mouse anti-Orb (4H8, 1:200, DHSB); chicken anti-GFP (1:1000, Aves GFP-1020); goat anti-Tj [1:7000, kind gift of Dorothea Godt (Li et al., 2003)]; rabbit anti-Zfh1 [1:5000, Fortini et al. (1991)]; rat anti-RFP (1:500, Chromotek 5F8); rabbit anti-Nanos (1:200, kind gift of Prof. Nakamura, Institute of Molecular Embryology and Genetics, Kumamoto University, Japan); rabbit anti-Aub [1:1000, Zamparini et al. (2011)]; rabbit anti-DsRed (1:500, Living Colors #632496). Alexa Fluor 647 Phalloidin- (1:500, Life Technologies) and rabbit anti-Myc Alexa Fluor 555- (Millipore 16-225) conjugated antibodies were used as secondary antibodies. Alexa Fluor 488- (Life Technologies), Cy3- or Alexa Fluor 647- (Jackson ImmunoResearch) conjugated secondary antibodies were used at 1:1000.

### Clone generation and quantification

*ptc>FLP;FRT82B,L(3)mbt^GM76^/FRT82B RFPnls* flies were raised at 25°C and shifted to 29°C after eclosion. In these conditions, mutant homozygous clones caused the *l(3)mbt* mutant phenotype in 6.7% of ovarioles (*n*=150) of 4- to 5-day-old females. Multiple wild-type twin spots were recovered in all ovarioles, suggesting loss of mutant clones.

For clones in larval ovaries, *c587>FLP;FRT82B,L(3)mbt^GM76^/FRT82B GFPnls* flies were raised at 29°C. A single medial *z* plane contained an average of 26±4 clones in the control experiment and 26.5±0.7*l(3)mbt^GM76^* clones. Control and *l(3)mbt* clones had comparable average sizes (basal clones: 32.74 and 30.54 square pixels, respectively, *P*=0.7; apical clones: 49.62 and 51.46, respectively; *P*=0.83, unpaired *t*-tests).

### Fluorescence intensity quantification in clones

Fluorescence intensity in the Vasa channel was measured in ten apical and ten basal wild-type and *l(3)mbt* mutant clones using FIJI/ImageJ (Schindelin et al., 2012) ROI manager function and normalized to the fluorescence intensity of identical, non-clonal ROIs.

### Quantification of the number of intermingled cells

*z*-stacks of five mutant and wild-type ovaries were acquired and Zfh1- or TJ-positive ICs were counted in a 75×30×13.5 µm compartment centered on the germ cell medial region using the Imaris software (v7.7.2) spot on detection and a spot size of 8 µm.

### RNA sequencing

Sixty to seventy ovaries from females of maternal *tud^1^/tud^B42^* and zygotic *l(3)mbt^GM76^/+* or *l(3)mbt^GM76^/Df* genotypes (see supplementary Materials and Methods) were dissected in cold PBS and RNA was extracted using TRIzol (Invitrogen) following the manufacturer's protocol. For early embryos, *TJ>UAS-l(3)mbt::myc;l(3)mbt^GM76^/+* and *TJ>UAS-l(3)mbt::myc;l(3)mbt^GM76^/Df* females were allowed to lay for 30 min to 1 h on agar plates. Embryos were dechorionated in 50% bleach for 5 min, rinsed with PBS, and then lysed in TRIzol. Libraries were generated from 1 µg of total RNA using the NEBNext Poly(A) magnetic Isolation Module (NEB #7490) and the NEBNext Ultra Directional RNA Library Prep Kit for Illumina (NEB #E7420). Libraries from biological replicates (three for ovaries, two for embryos) were sequenced on an Illumina Hi-Seq2000, paired-end 50 run.

### RNA-seq data analysis

Sequencing results were demultiplexed and converted to FASTQ format using Illumina Bcl2FastQ software (Illumina). Reads were aligned to the fly genome (build dm3/BDGP5) using the splice-aware STAR aligner (Dobin et al., 2013). PCR duplicates were removed using the Picard toolkit (https://broadinstitute.github.io/picard/). The HTSeq package was used to generate counts for each gene based on how many aligned reads overlap its exons (Anders et al., 2015). These counts were then used to test for differential expression using negative binomial generalized linear models implemented by the DESeq2 R package.

### Single-molecule RNA FISH

*TJ>UAS-nos; Gal80^ts^/UAS-mCherry-myr* and control *+/ UAS-nos; Gal80^ts^/UAS-mCherry-myr* ovaries were stained as previously described (Trcek et al., 2017). Briefly, ten pairs of ovaries of flies switched at 29°C after eclosion were dissected, fixed for 20 min in 4% PFA in PBS, permeabilized at 4°C in methanol overnight, rehydrated and post-fixed for 20 min in 4% PFA in PBS, and hybridized overnight in hybridization solution containing a mixture of commercially available Stellaris smFISH probes each labeled with a Quasar 670 fluorophore. Each smFISH probe in the mixture was designed to anneal to a different position within the open reading frame of *nos* mRNA thereby significantly increasing signal-to-noise ratio during imaging and single molecule detection (Trcek et al., 2017).

### Quantification of the number of *nanos* RNA molecules in follicle cells

The absolute number of *nos* RNA molecules was quantified using Airlocalize software (Lionnet et al., 2011; Trcek et al., 2017). Briefly, molecules were counted in a single medial *z*-plane using an ROI of 68×102 pixels approximately corresponding to the size of a wild-type follicle cell.

### Fertility test of *Lint-1^1^* females

Fifteen *Lint-1^1^* homozygous females were crossed to wild-type males in vials for 10 days at 25°C in duplicate. No eggs resulting from this cross hatched.

### Tissue expression clustering

Expression of deregulated genes was extracted from FlyAtlas (Chintapalli et al., 2007) using FlyBase IDs, normalized to fly average and log_2_ transformed. Distance matrix was calculated using the ‘Manhattan’ method and data clustered using ‘ward.D2’. Heatmap was generated using the heatmap.2 function of the gplots R package.

## Supplementary Material

Supplementary information

Supplementary information
