# Supplementary material for: L(3)mbt and the LINT complex safeguard cellular identity in the Drosophila ovary
Source: Development. 2018 Apr 4;145(7):dev160721. doi: 10.1242/dev.160721 (PMC5963868; doi:10.1242/dev.160721)
Supplement: Supplementary information [file develop-145-160721-s1.pdf]

Supplementary Materials and Methods

Genotypes

Name	Genotype	Figure	Origin
L(3)mbt^{GM76}*/+*	*P[neoFRT]82B l(3)mbt*^{GM76} *e/TM6b*	1, 3, 4, S1, S3, S4	(Richter et al., 2011; Yohn et al., 2003)
L(3)mbt^{GM76}*/Df*	*P[neoFRT]82B l(3)mbt*^{GM76} *e /Df(3R)ED19066*	1, 3, 4, S1, S3, S4	Kyoto #150208
Ptc>FLP; FRT82B l(3)mbt^{GM7}*/FRT82B RFPnls*	*P{UAS-FLP.D}JD1 /P{GawB}ptc*$^{559.1}$*; P[neoFRT]82B l(3)mbt*^{GM76} *e/ P[neoFRT]82B P[Ubi-mRFP.nls]*	2A, 2B	BDSC #4539, 2017, 30555
C587>FLP;; FRT82B l(3)mbt^{GM76}*/FRT82B GFPnls*	*c587-Gal4,UAS-FLP;; P[neoFRT]82B, l(3)mbt*^{GM76} *e / P[neoFRT]82B P[Ubi-GFPnls]*	3D	(Zhu and Xie, 2003)<br>BDSC #32655
C587>FLP;; FRT-control/FRT GFPnls	*c587-Gal4,UAS-FLP;; P[neoFRT]82B, ry*^{506}*/ P[neoFRT]82B P[Ubi-GFPnls]*	3C	BDSC #32655<br>BDSC #225
TJ>l(3)mbt::myc; L(3)mbt^{GM76}*/Df*	*P{UAS-lacZ.UW14}UW14/P[UASp-l(3)mbt::myc]; P[neoFRT]82B l(3)mbt*^{GM76} *e /Df(3R)ED19066*	2C,D, S2 and 8-S8	Kyoto #104055 and this study
tud^{M}*; l(3)mbt*^{GM76}*/Df* (females devoid of germline)	**Maternal genotype:** *tud*^{1}*/tud*^{B42}*; P[neoFRT]82B l(3)mbt*^{GM76} *e /TM6b* or *tud*^{1}*/tud*^{B42}*; Df(3R)ED19066/TM6b*<br>**Paternal genotype:** *+; P[neoFRT]82B l(3)mbt*^{GM76} *e /TM6b* or *+; Df(3R)ED19066/TM6b*<br>**Zygotic genotype:** *tud/+; P[neoFRT]82B l(3)mbt*^{GM76} *e /Df(3R)ED19066*<br>Control: *tud/Cyo; P[neoFRT]82B l(3)mbt*^{GM76} *e /TM6b*	4 and S4 (somatic ovary sequencing)	(Arkov et al., 2006)
nos, l(3)mbt	*P[neoFRT]82B l(3)mbt*^{GM76} *e nos*^{L7}*/ P[neoFRT]82B l(3)mbt*^{GM76} *e nos*^{BN}<br>Control:<br>*P[neoFRT]82B l(3)mbt*^{GM76} *e nos*^{L7}*/ P[neoFRT]82B l(3)mbt*^{GM76} *e*	5A-E	(Wang and Lehmann, 1991; Wang et al., 1994)

pum, l(3)mbt	*P[neoFRT]82B l(3)mbt*^{GM76} *e pum*^{680}*/* *P[neoFRT]82B l(3)mbt*^{GM76} *e pum*^{680}<br>Control:<br>*P[neoFRT]82B l(3)mbt*^{GM76} *e pum*^{680}*/* *P[neoFRT]82B l(3)mbt*^{GM76} *e*	5F-H	(Lehmann and Nüsslein-Volhard, 1987)
C587>FLP;; FRT40A E2F2^{c03344}*/FRT40A GFP*	*c587-Gal4 UAS-FLP; E2F2*^{c03344} *P[neoFRT]40A /P{Ubi-GFP.D}33 P{ Ubi-GFP.D}38 P[neoFRT]40A*	7C	(Ambrus et al., 2007)
TdTomato::L(3)mbt; mip$^{12067.9A.9}$*/+*	*TdTomato::L(3)mbt; mip*$^{12067.9A.9}$*/Cyo*	S7	(Beall et al., 2007; Blanchard et al., 2014)
TdTomato::L(3)mbt; mip$^{12067.9A.9/12067.9A.9}$	*TdTomato::L(3)mbt; mip*$^{12067.9A.9}$*/ mip*$^{12067.9A.9}$	S7	(Beall et al., 2007; Blanchard et al., 2014)
Lint1^{1}*/Lint1*^{1}	*Lint1*^{1} *P[neoFRT]19A / Lint1*^{1} *P[neoFRT]19A*	7E and F	This study
aub^{HN2}*/aub*^{QC42}*; L(3)mbt*^{GM76}*/l(3)mbt*^{GM76}	*aub*^{HN2}*cn bw/aub*^{HN2}*cn bw; P[neoFRT]82B l(3)mbt*^{GM76} *e / P[neoFRT]82B l(3)mbt*^{GM76} *e*	S5	(Schupbach and Wieschaus, 1991)
TJG4>UAS-nos; Gal80ts/UAS myr-mCherry	*P{UAS-lacZ.UW14}UW14/P[UAS-nos-tub-3'UTR]; P{tubP-GAL80*^{ts}*}2/P[UAS myr-mCherry]*	6-S6A	(Clark et al., 2002)BDSC #7017<br>Zallen lab
TJ>UAS-aubGFP	*P{UAS-lacZ.UW14}UW14/P[UAS-aub-GFP]*	S6C	(Harris and Macdonald, 2001)
TJ>UAS-vas	*P{UAS-lacZ.UW14}UW14/P[UAS-vas]*	S6D	(Sengoku et al., 2006)

Molecular nature of alleles

L(3)mbt^{GM76} is a nonsense mutation (W928term), the truncated protein lacks a C-terminus moiety including two out of three MBT domains and is unable to bind DNA (Blanchard et al., 2014).

Aub^{HN2} is a nonsense mutation (Q622term) and the precise nature of *aub*^{QC4} remains unknown.

Nos^{BN} results from a P-element insertion in *nos* 5' region resulting in proper *nos* expression during early to mid-oogenesis but not at later stages (Wang et al., 1994).

Nos^{L7} deletes nucleotides 2009-2029 in Nos C terminus, disrupting Pum binding (Sonoda and Wharton, 1999).

Pum^{680} induces a single nucleotide substitution in Pum C-terminal RNA binding domain (G1330D) (Wharton et al., 1998)

E2F2^{c03344} is a Piggyback insertion in *E2F2* (Ambrus et al., 2007)

Mip120$^{67.9A9}$ results from the imprecise excision of a P-element deleting most of the coding sequence (Beall et al., 2007)

References

Ambrus, A. M., Nicolay, B. N., Rasheva, V. I., Suckling, R. J. and Frolov, M. V. (2007). dE2F2-independent rescue of proliferation in cells lacking an activator dE2F1. *Mol Cell Biol* **27**, 8561–8570.

Arkov, A. L., Wang, J.-Y. S., Ramos, A. and Lehmann, R. (2006). The role of Tudor domains in germline development and polar granule architecture. *Development* **133**, 4053–4062.

Beall, E. L., Lewis, P. W., Bell, M., Rocha, M., Jones, D. L. and Botchan, M. R. (2007). Discovery of tMAC: a Drosophila testis-specific meiotic arrest complex paralogous to Myb-Muv B. *Genes & Development* **21**, 904–919.

Blanchard, D. P., Georlette, D., Antoszewski, L. and Botchan, M. R. (2014). Chromatin reader L(3)mbt requires the Myb-MuvB/DREAM transcriptional regulatory complex for chromosomal recruitment. *Proc Natl Acad Sci USA*.

Clark, I. E., Dobi, K. C., Duchow, H. K., Vlasak, A. N. and Gavis, E. R. (2002). A common translational control mechanism functions in axial patterning and neuroendocrine signaling in Drosophila. *Development* **129**, 3325–3334.

Harris, A. N. and Macdonald, P. M. (2001). Aubergine encodes a Drosophila polar granule component required for pole cell formation and related to eIF2C. *Development* **128**, 2823–2832.

Lehmann, R. and Nüsslein-Volhard, C. (1987). Involvement of the pumilio gene in the transport of an abdominal signal in the Drosophila embryo. *Nature* **329**, 167–170.

Richter, C., Oktaba, K., Steinmann, J. and Knoblich, J. A. (2011). The tumour suppressor L(3)mbt inhibits neuroepithelial proliferation and acts on insulator elements. *Nat Cell Biol* **13**, 1029–1039.

Schupbach, T. and Wieschaus, E. (1991). Female sterile mutations on the second chromosome of Drosophila melanogaster. II. Mutations blocking oogenesis or altering egg morphology. *Genetics* **129**, 1119–1136.

Sengoku, T., Nureki, O., Nakamura, A., Kobayashi, S. and Yokoyama, S. (2006). Structural basis for RNA unwinding by the DEAD-box protein Drosophila Vasa. *Cell* **125**, 287–300.

Sonoda, J. and Wharton, R. P. (1999). Recruitment of Nanos to hunchback mRNA by Pumilio.

Genes & Development **13**, 2704–2712.

Wang, C. and Lehmann, R. (1991). Nanos is the localized posterior determinant in Drosophila. *Cell* **66**, 637–647.

Wang, C., Dickinson, L. K. and Lehmann, R. (1994). Genetics of nanos localization in Drosophila. *Dev Dyn* **199**, 103–115.

Wharton, R. P., Sonoda, J., Lee, T., Patterson, M. and Murata, Y. (1998). The Pumilio RNA-binding domain is also a translational regulator. *Mol Cell* **1**, 863–872.

Yohn, C. B., Pusateri, L., Barbosa, V. and Lehmann, R. (2003). l(3)malignant brain tumor and three novel genes are required for Drosophila germ-cell formation. *Genetics* **165**, 1889–1900.

Zhu, C.-H. and Xie, T. (2003). Clonal expansion of ovarian germline stem cells during niche formation in Drosophila. *Development* **130**, 2579–2588.

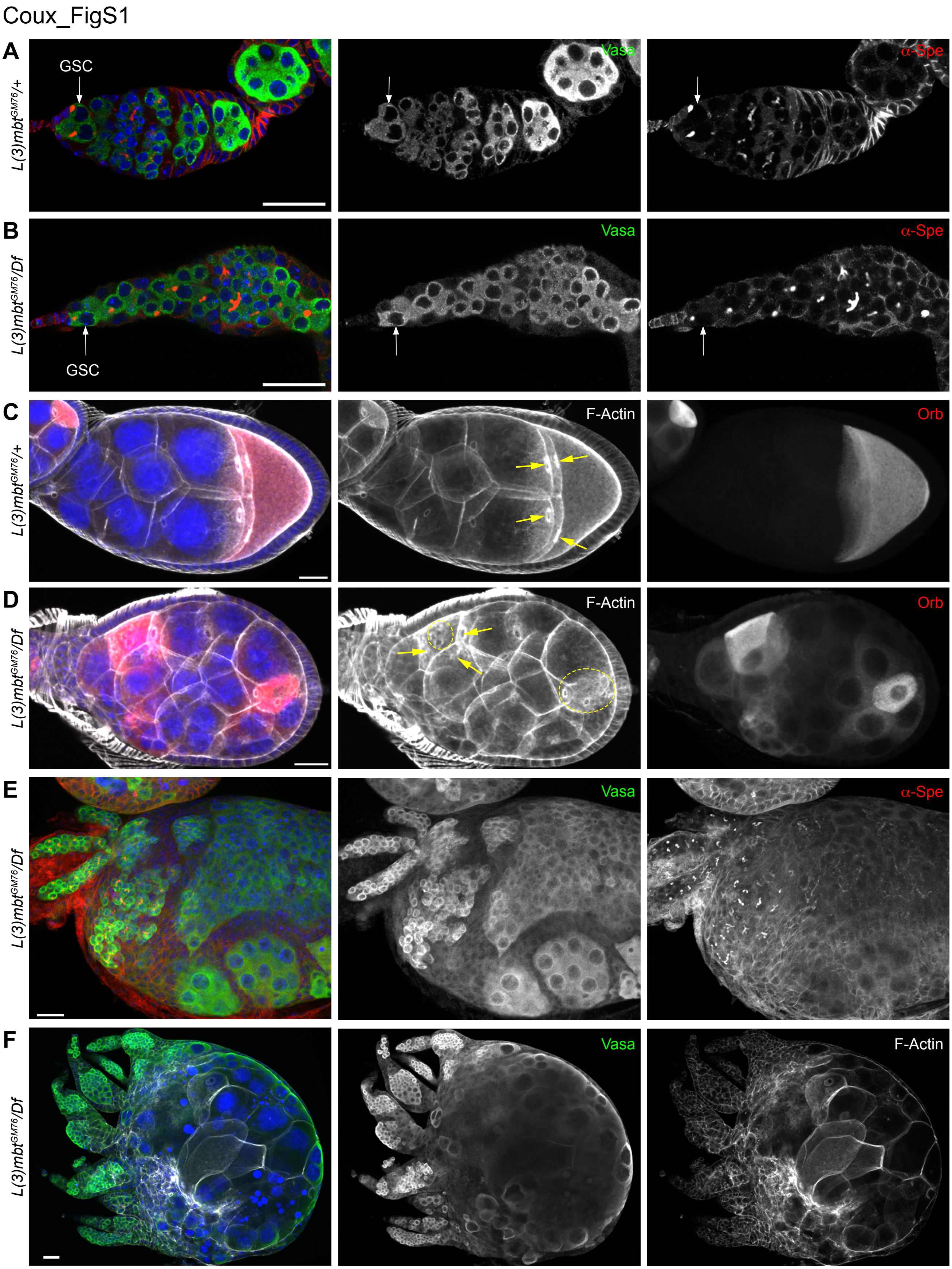
Coux_FigS1
A
L(3)mbt^GM76/+
GSC
Vasa
α-Spe
B
L(3)mbt^GM76/Df
GSC
Vasa
α-Spe
C
L(3)mbt^GM76/+
F-Actin
Orb
D
L(3)mbt^GM76/Df
F-Actin
Orb
E
L(3)mbt^GM76/Df
Vasa
α-Spe
F
L(3)mbt^GM76/Df
Vasa
F-Actin

Supplementary figure 1. L(3)mbt loss-of-function leads to egg chamber and ovariole fusions. *(A-B)* Confocal images of wild-type *(A)* and mutant *(B)* germaria, stained for Vasa (green), α-Spectrin (red) and DAPI (blue). Wild-type and mutant germaria contain Germline Stem Cells marked by punctuated fusomes (α-Spectrin), in contact with the somatic niche. *(C-D)* Confocal projections of (*C)* wild-type and (*D)* mutant ovaries stained for F-Actin (grey), Orb (red), and DAPI (blue). Anterior is oriented to the left. (C) a wild-type oocyte is connected to nurse cells by four ring canals (yellow arrows). (*D*) *L(3)mbt* mutant egg chamber containing multiple oocytes with four or more ring canals (arrows and dotted circles). (*E)* Confocal image of *l(3)mbt* mutant ovary stained for Vasa (green), α-Spectrin (red) and DAPI (blue). Three germaria (top left) fused into an aberrant ovariole with intermingled differentiated and undifferentiated germ cells. *(F) l(3)mbt* mutant ovary with multiple germaria connected to the same mass of egg chamber. Vasa (green), F-Actin (grey) and DAPI (blue). Scale bars, 25 μm.

Coux_FigS2

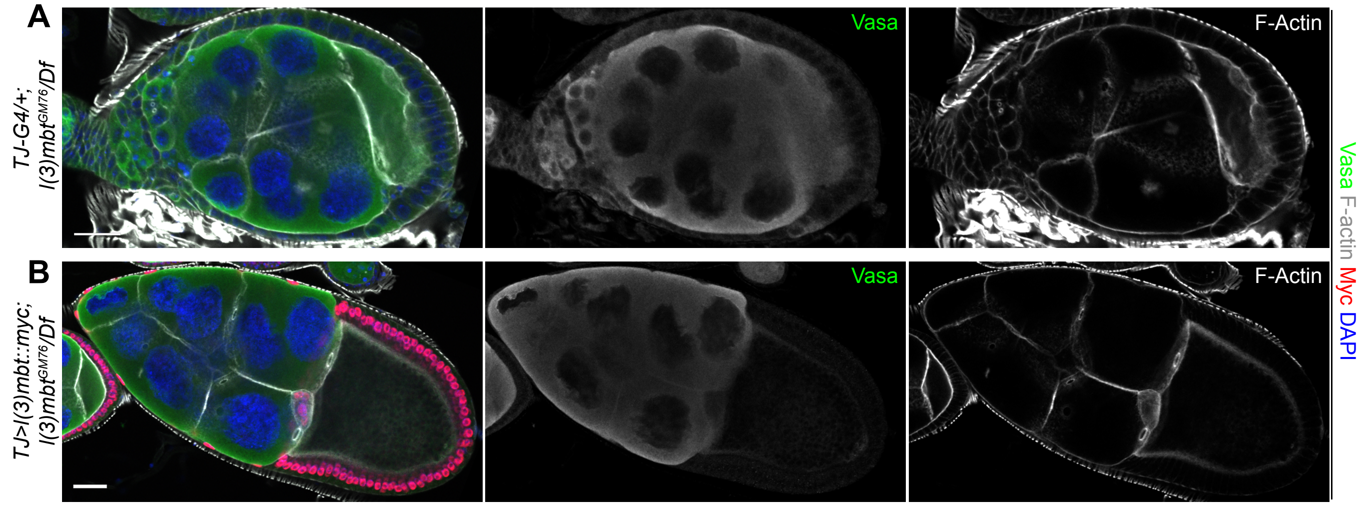

Supplementary figure 2. L(3)mbt somatic expression rescues *l(3)mbt* mutant ovarian morphology. Confocal images of ovarioles from (*A*) *l(3)mbt* mutant control and (*B*) *l(3)mbt* mutant expressing the *l(3)mbt::myc* transgene in somatic cells, stained for Vasa (green), Myc (red), F-Actin (grey), and DAPI (blue). Complemented ovarioles show wild-type morphology, including proper oocyte specification and ring canals numbers. Scale bars, 25 µm.

Development • Supplementary information

Coux_FigS3

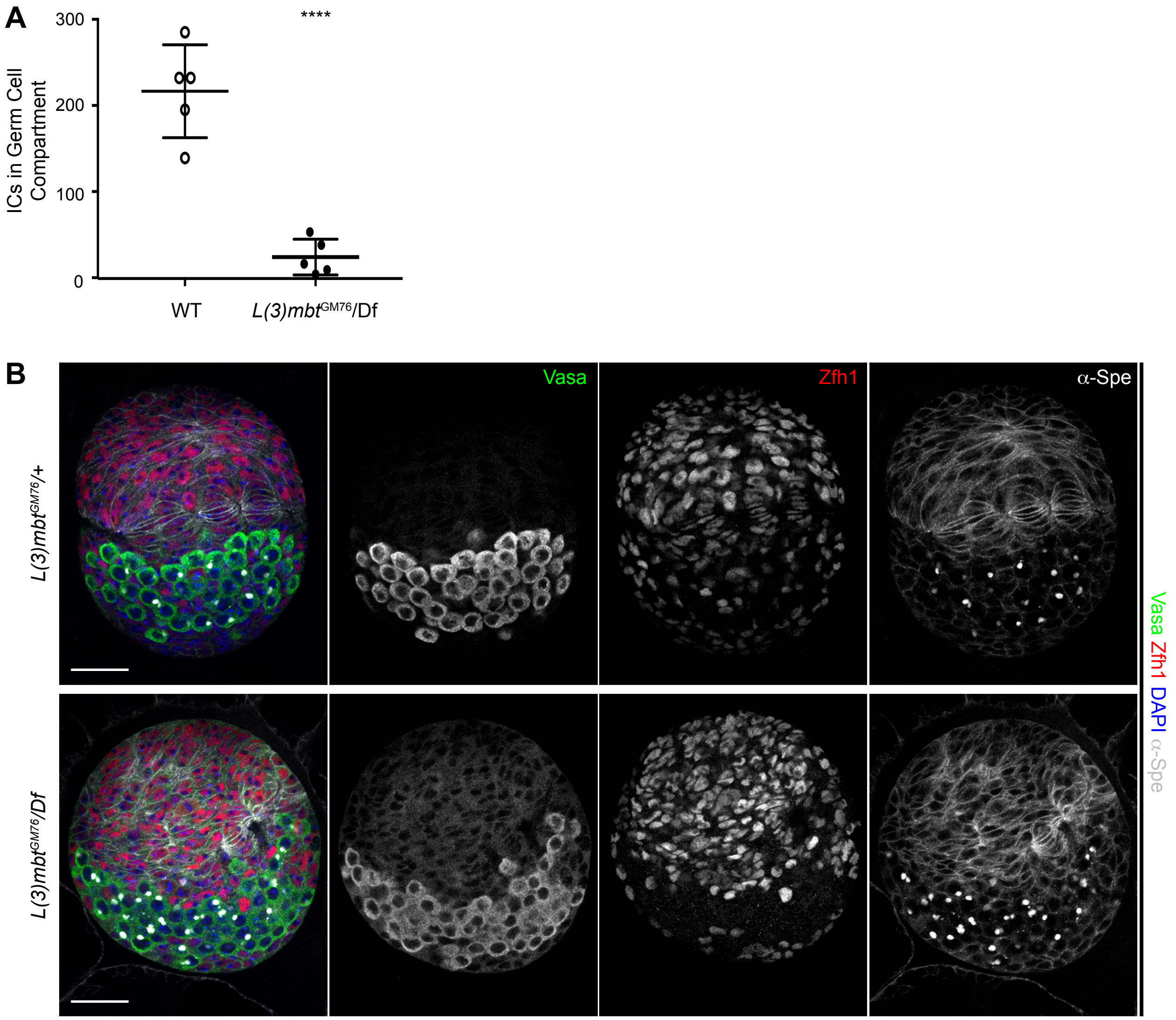

Supplementary figure 3. *(A)* Quantification of the number of Intermingled Cells in an arbitrarily defined germ cell compartment in wild-type and *l(3)mbt* mutant L3 ovaries. ****, $p<10^{-4}$, unpaired t-test. (B) *L(3)mbt* mutant somatic larval cells have normal Zfh1 expression. Wild-type (top panel) and *l(3)mbt* mutant (bottom). L3 ovaries stained for vasa (green), α-spectrin (grey), Zfh1 (red) and DAPI (blue). Scale bars represent 25μm.

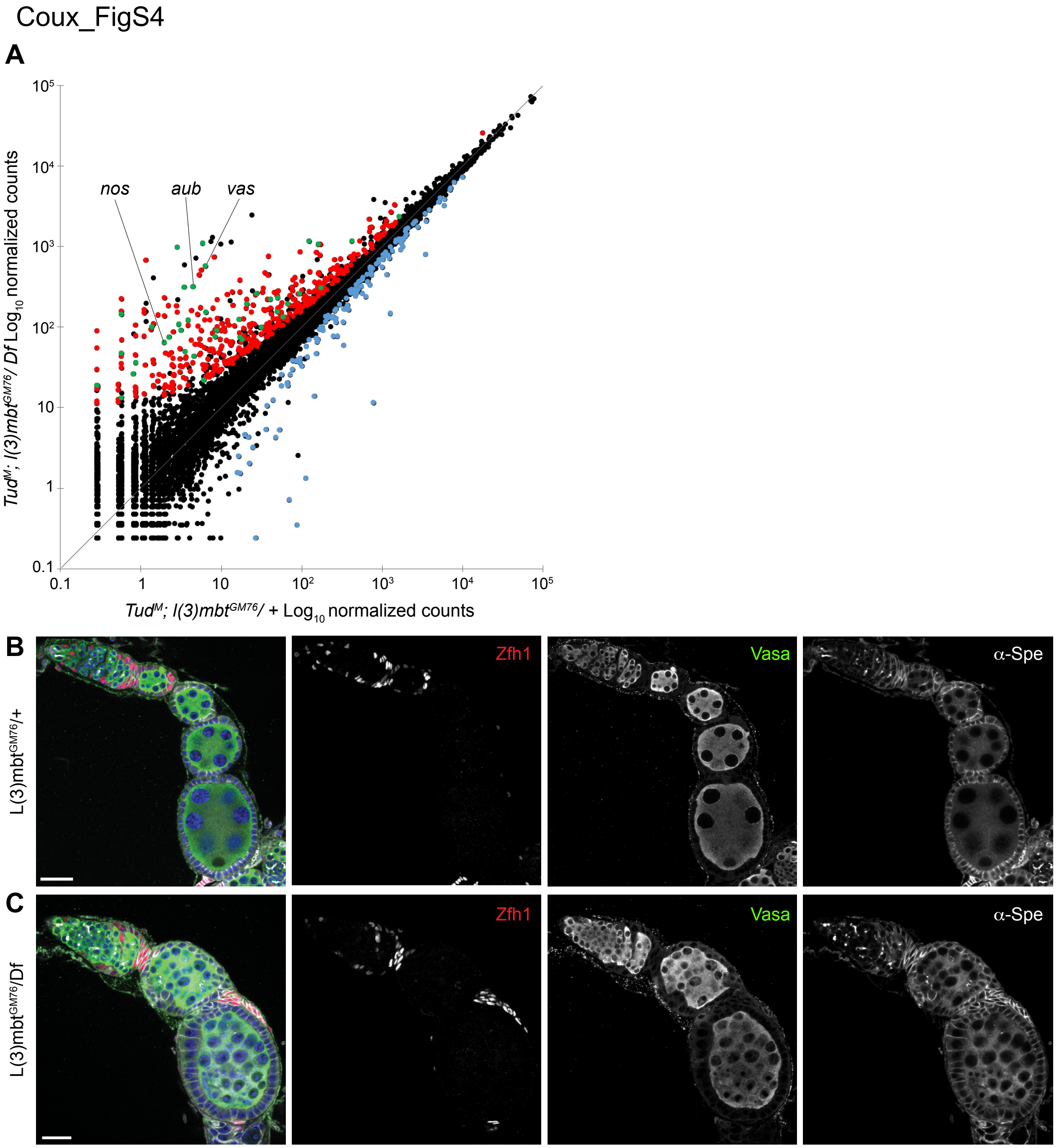

Supplementary figure 4. *L(3)mbt* mutant somatic cells are properly specified but ectopically express germline genes. *(A)* Scatterplot showing the expression of genes in *tud*^{M}; *l(3)mbt*^{GM76}/+ and *tud*^{M}; *l(3)mbt*^{GM76} mutant ovaries, as measured by RNA-seq (normalized counts, $\log_{10}$). Upregulated genes are shown in red, downregulated in blue and MBTS genes in green. (*B*-*C*) Confocal images of (*B*) control and (*C*) *l(3)mbt* mutant ovarioles stained for Vasa (green), Zfh1 (red), α-Spectrin (grey), and DAPI (blue). (*B*) In control ovaries, Zfh1 is expressed in escort cells, pre-follicle cells as well as stalk cells, which separate egg chambers. *(C) L(3)mbt* mutant ovariole showing normal Zfh1 expression but stalk cells accumulate on top of follicle cells. Scale bars, 25 µm.

Coux_FigS5

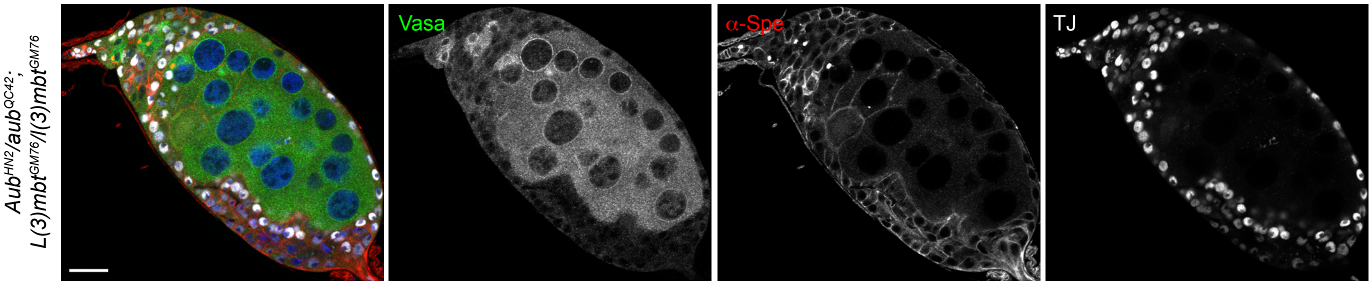

Supplementary figure 5. *Aub* ectopic expression is not required for *l(3)mbt* ovarian phenotype. Confocal image of *aub; l(3)mbt* double mutant ovariole stained for Vasa (green), α-Spectrin (red); TJ (grey), and DAPI (blue). Scale bar, 25 μm. The phenotype is similar to single *l(3)mbt* mutant.

Development • Supplementary information

Coux_FigS6

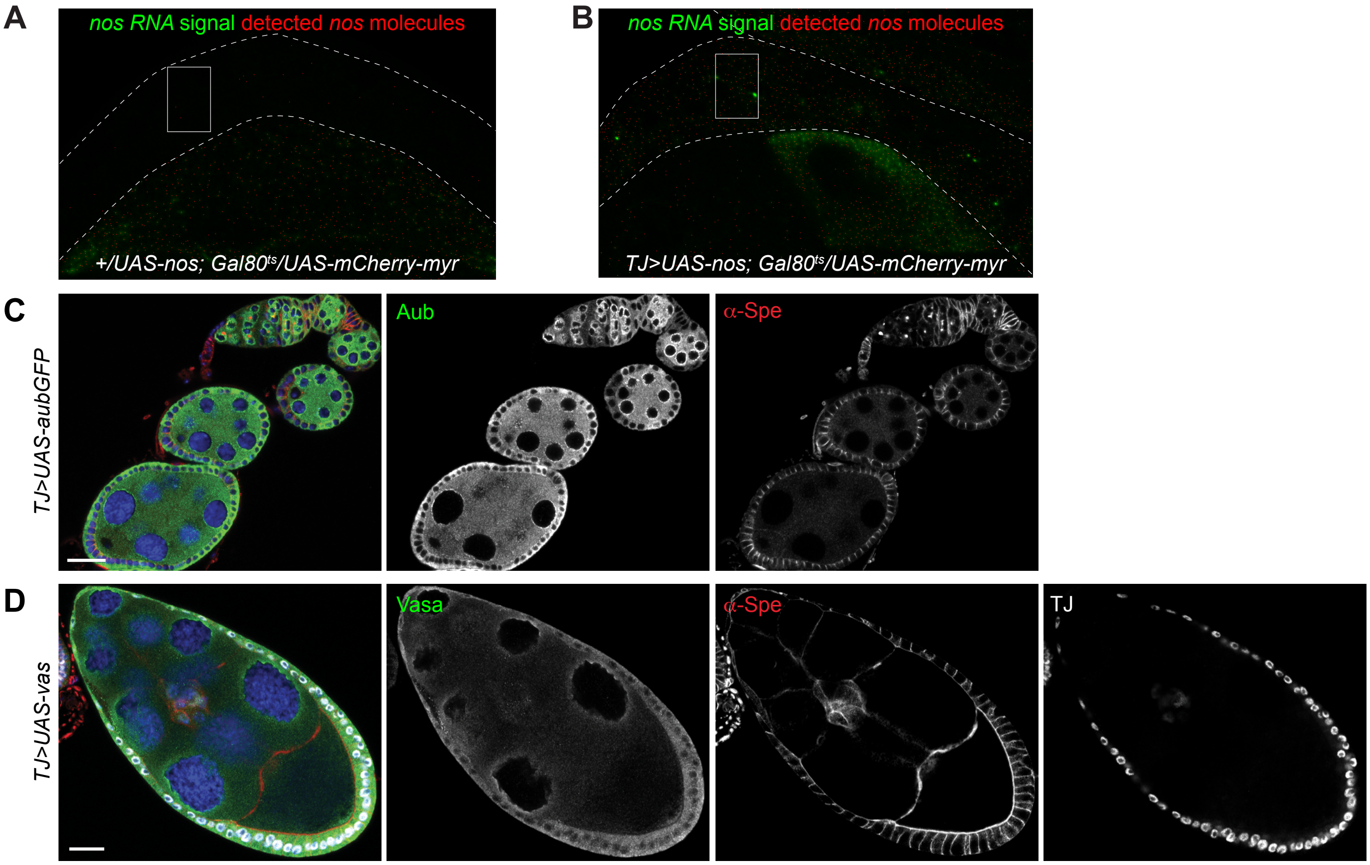

Supplementary figure 6. *Nos*, but not *Aub* or *Vas* ectopic expression interferes with normal ovarian development. *(A-B)* Representatitve images post *nos* smRNA FISH quantification using Airlocalize, *nos* RNA is shown in green and detected molecules as red spots. The layer of somatic follicle cells is outlined in dotted lines and the ROI used for quantification is shown as a white rectangle. *(C-D)* Confocal images of ovaries misexpressing Aub or Vasa in somatic cells using the TJ-Gal4 driver and the (*C*) UAS-aubGFP and (*D*) UAS-vas transgenes. Ovaries were stained for (*C*) Aub (green), α-Spectrin (red), and DAPI (blue), or (*D*) Vasa (green), α-Spectrin (red), and TJ (grey). Scale bars, 25 μm.

Development • Supplementary information

Coux_FigS7

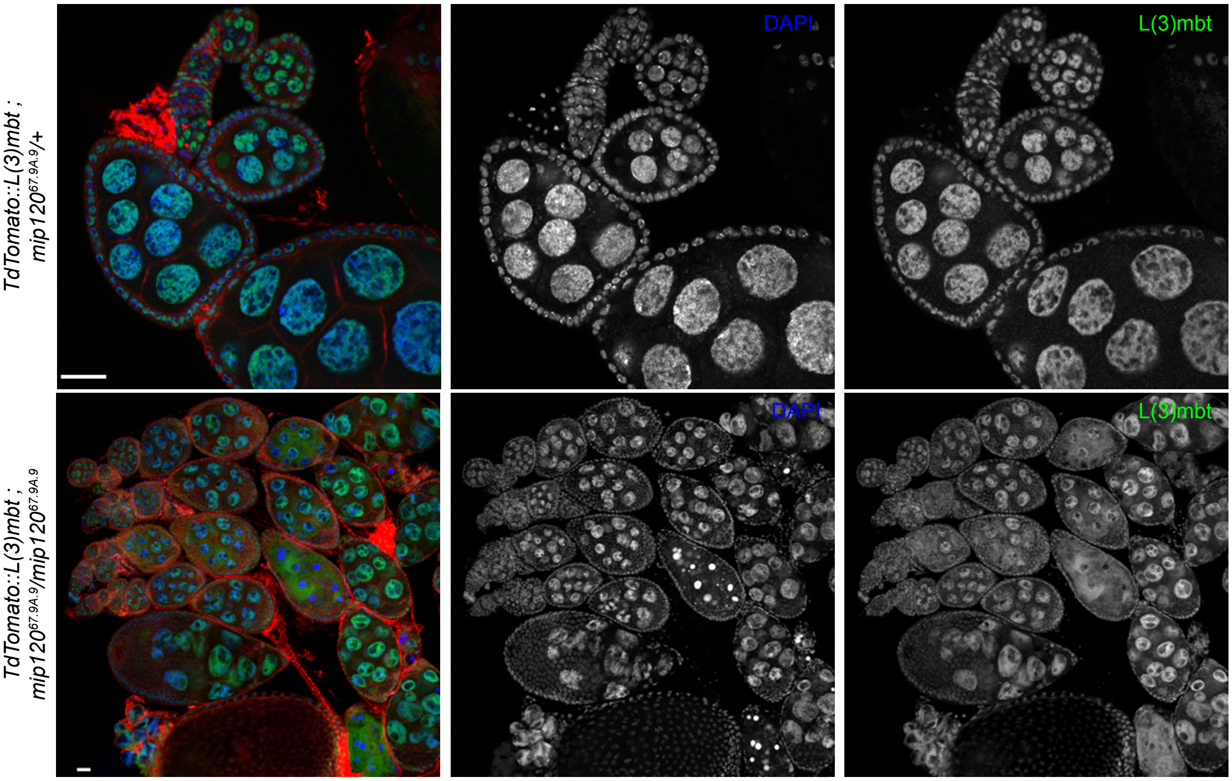

Supplementary figure 7. Confocal images of control and *mip120*$^{67A9.9}$ mutant ovaries expressing the tdTomato::L(3)mbt fusion and stained for α-Spectrin (red), tdTomato (green), and DAPI (blue). Top panel, *mip120*$^{67A9.9}$ heterozygous control ovarioles. Bottom panel, low magnification confocal image of homozygous mutant *mip120*$^{67A9.9}$ ovarioles accumulating stage 7-9 egg chambers that eventually undergo apoptosis. Scale bars, 25 µm.

Development • Supplementary information

Coux_FigS8

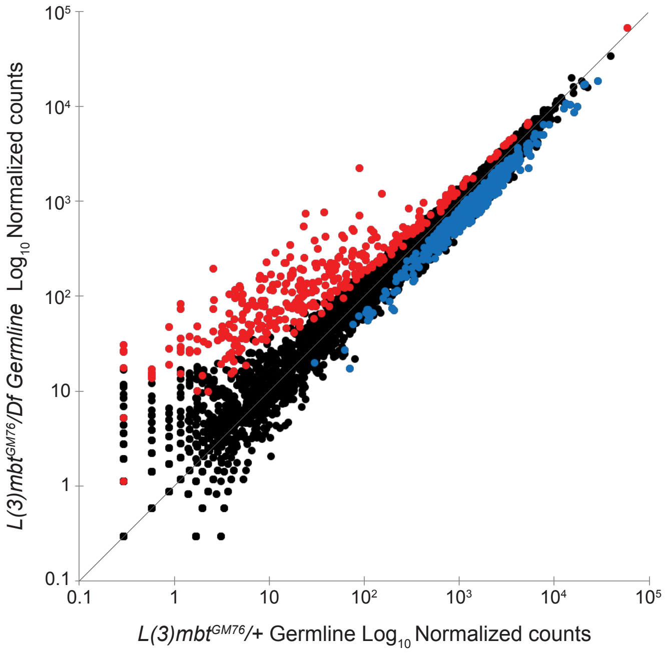

Supplementary figure 8. Scatterplots showing expression of genes in control and *l(3)mbt*^{GM76} mutant early embryos, as measured by RNA-seq (normalized counts, log_{10}). Up-regulated genes are shown in red, down-regulated genes are shown in blue.

Supplementary Table 1. Genes differentially expressed (diff_exp_q<0.05) and normalized counts in *l(3)mbt* mutant somatic ovaries (*tud*^{M}*; l(3)mbt*^{GM76}*/Df* compared to *tud*^{M}*; l(3)mbt*^{GM76}/+). BaseMean corresponds to the average of the normalized counts taken over all samples, Log2FC is Log2 Fold Change mutant/control, pvalue is the Wald test p-value and padj, Benjamini-Hochberg adjusted p-value.

Click here to Download Table S1

Supplementary Table 2. Genes differentially expressed (diff_exp_q<0.05) and normalized counts in embryos laid by *l(3)mbt* mutant germline *(TJ>l(3)mbt::myc; L(3)mbt*^{GM76}*/Df*, control *TJ>l(3)mbt::myc; L(3)mbt*^{GM76}/+). (BaseMean corresponds to the average of the normalized counts taken over all samples, Log2FC is Log2 Fold Change mutant/control, pvalue is the Wald test p-value and padj, Benjamini-Hochberg adjusted p-value.

Click here to Download Table S2

Supplementary Table 3. Oligo nucleotide sequences used for *lint-CRISPR* (described in Figure 7) and RNA FISH experiments (described in Figure 6).

**Experiment**	**Oligo Name**	**Sequence**
Lint1 CRISPR	X:11044844-11044866_R	CTTCGACGATCAAAGGCACTACCT
	X: 11044844-11044866_F	AAAC CCCAGGTAGTGCCTTTGATCGTC
Nos smRNA FISH	5'_1	tccaagttgctgcggaacat
	5'_2	tactgctgctgcgccactgc
	5'_3	acgaggggggatttgcaaca
	5'_4	aaaatctttccagactgagc
	5'_5	agaaaagttatcctgcaatt
	5'_6	gccctcctctggcgtgaaaa
	5'_7	tcctgcaggcccagaatgtt
	5'_8	cccactggtatccaaataca
	5'_9	tcaaagtggccgacgagttg
	5'_10	aggggtcaccggcgtaatgg
	5'_11	actgcgcagacgtcgacggg
	5'_12	gccagaaaagggaagtgcgt
	5'_13	attggcggtggctgcgctgt
	5'_14	actgtcgctgcataaggagc
	5'_15	tggagcagcaagtggtagtg
	5'_16	catggccagttgctgctgct
	5'_17	ccagcgccaattggtgctgc
	5'_18	ctagccgctgccgctgatgc
	5'_19	gtccgtttgctggtgactcg
	5'_20	ttttcaaggatcgcgcaatc
	5'_21	cccgtcgtcacctgcgcaaa
	5'_22	agccagccgcattttctgct
	5'_23	tcctgcatcacatcctgcat
	5'_24	ggcatagccattggtcgcga
	5'_25	acatgcgaccgagatcatcg
	5'_26	tgtggcggagcactcccgta
	5'_27	ctgctgcggtggcatttgca
	**3'_1**	agcccctgctgttgctgatg
	**3'_2**	attgcggcccagtggcaggt
	**3'_3**	cattggtctgcagctgggca
	**3'_4**	tggaatgggcattaagttgc
	**3'_5**	tgttcagccagtgggtggcg
	**3'_6**	ttgttcagatgctcccggta
	**3'_7**	atacgacatgtttcgccaca
	**3'_8**	ttgtattgggagcggctggt
	**3'_9**	gctgtttgggcctgcaaacc
	**3'_10**	gagattggtggacacagtgg
	**3'_11**	atcccagacccattcccacg
	**3'_12**	agctgttcgccctgcacggg
	**3'_13**	actggaattggaagctccgc
	**3'_14**	tgttgttgttgttattattg
	**3'_15**	ctgttgtaacgcttgtacac
	**3'_16**	gcggctgatctctttggcct
	**3'_17**	tattctcacaaaagacgcag

	**3'_18**	ataaccgcctctggttcgtt
	**3'_19**	atctcgcactgagtggctat
	**3'_20**	ggcacagcactcggttaaag
	**3'_21**	cacacgtaggtgcgtagttt
	**3'_22**	tccccagatgccccgcagat
	**3'_23**	agtacttaatcgtgtgcgcc
	**3'_24**	atggtgatgatcggcttctt
	**3'_25**	ttccgccttgatcgcatcct
	**3'_26**	taactgctcttggctaggcg
	**3'_27**	aaccttcatctgttgcttgt

Development • Supplementary information
